# Dissociated Oxygen Consumption and Carbon Dioxide Production in the Post–Cardiac Arrest Rat: A Novel Metabolic Phenotype

**DOI:** 10.1161/JAHA.117.007721

**Published:** 2018-06-29

**Authors:** Koichiro Shinozaki, Lance B. Becker, Kota Saeki, Junhwan Kim, Tai Yin, Tong Da, Joshua W. Lampe

**Affiliations:** ^1^ Feinstein Institute for Medical Research Northwell Health System Manhasset NY; ^2^ ZOLL Medical Chelmsford MA; ^3^ Nihon Kohden Innovation Center, INC. Cambridge MA; ^4^ Center for Cellular Immunotherapies The University of Pennsylvania Philadelphia PA

**Keywords:** CO_2_ production, cardiopulmonary resuscitation (CPR), metabolism, O_2_ consumption, respiratory quotient, Translational Studies, Cardiopulmonary Arrest, Cardiopulmonary Resuscitation and Emergency Cardiac Care

## Abstract

**Background:**

The concept that resuscitation from cardiac arrest (CA) results in a metabolic injury is broadly accepted, yet patients never receive this diagnosis. We sought to find evidence of metabolic injuries after CA by measuring O_2_ consumption and CO_2_ production (VCO_2_) in a rodent model. In addition, we tested the effect of inspired 100% O_2_ on the metabolism.

**Methods and Results:**

Rats were anesthetized and randomized into 3 groups: resuscitation from 10‐minute asphyxia with inhaled 100% O_2_ (CA–fraction of inspired O_2_ [FIO_2_] 1.0), with 30% O_2_ (CA‐FIO_2_ 0.3), and sham with 30% O_2_ (sham‐FIO_2_ 0.3). Animals were resuscitated with manual cardiopulmonary resuscitation. The volume of extracted O_2_ (VO_2_) and VCO_2_ were measured for a 2‐hour period after resuscitation. The respiratory quotient (RQ) was RQ=VCO_2_/VO_2_. VCO_2_ was elevated in CA‐FIO_2_ 1.0 and CA‐FIO_2_ 0.3 when compared with sham‐FIO_2_ 0.3 in minutes 5 to 40 after resuscitation (CA‐FIO_2_ 1.0: 16.7±2.2, *P*<0.01; CA‐FIO_2_ 0.3: 17.4±1.4, *P*<0.01; versus sham‐FIO_2_ 0.3: 13.6±1.1 mL/kg per minute), and then returned to normal. VO_2_ in CA‐FIO_2_ 1.0 and CA‐FIO_2_ 0.3 increased gradually and was significantly higher than sham‐FIO_2_ 0.3 2 hours after resuscitation (CA‐FIO_2_ 1.0: 28.7±6.7, *P*<0.01; CA‐FIO_2_ 0.3: 24.4±2.3, *P*<0.01; versus sham‐FIO_2_ 0.3: 15.8±2.4 mL/kg per minute). The RQ of CA animals persistently decreased (CA‐FIO_2_ 1.0: 0.54±0.12 versus CA‐FIO_2_ 0.3: 0.68±0.05 versus sham‐FIO_2_ 0.3: 0.93±0.11, *P*<0.01 overall).

**Conclusions:**

CA altered cellular metabolism resulting in increased VO_2_ with normal VCO_2_. Normal VCO_2_ suggests that the postresuscitation Krebs cycle is operating at a presumably healthy rate. Increased VO_2_ in the face of normal VCO_2_ suggests a significant alteration in O_2_ utilization in postresuscitation. Several RQ values fell well outside the normally cited range of 0.7 to 1.0. Higher FIO_2_ may increase VO_2_, leading to even lower RQ values.

**Clinical Perspective**

**What Is New?**

- Cardiac arrest altered cellular metabolism resulting in increased O_2_ consumption with normal exhaled CO_2_ in a rodent model.
- Increased O_2_ consumption in the face of normal exhaled CO_2_ suggests a significant alteration in O_2_ utilization in postresuscitation.
- Our calculated respiratory quotient values, well outside the normally cited range of 0.7 to 1.0 have been barely reported in previous reports.

**What Are the Clinical Implications?**

- Higher concentration of inspired O_2_ may increase O_2_ consumption, leading to even lower respiratory quotient values.
- Measuring O_2_ consumption and exhaled CO_2_ through mechanical ventilation is noninvasive and our calculated respiratory quotient values warrant larger animal and human studies.

## Introduction

Exciting data from our laboratory have demonstrated that our current understanding of aerobic respiration cannot explain the O_2_ consumption (VO_2_) measured in rats resuscitated from prolonged cardiac arrest (CA), suggesting that we have discovered a new postresuscitation metabolic phenotype.

CA is a time‐dependent pathology,^1^ and the majority of patients with CA are found more than 10 minutes after their arrest occurred.^2^, ^3^ Patients with long untreated arrest times have the worst outcomes,^2^, ^3^, ^4^ in part because there is much to learn about the injury sustained by patients resuscitated from prolonged CA. The loss of blood flow caused by CA is widely acknowledged to cause a metabolic pathology, but we struggle to measure the effect of this pathology at the bedside. The cessation of blood flow during CA stops delivery of metabolic substrates and removal of metabolic waste products, rapidly causing a variety of metabolic disorders including loss of ATP, acidemia, hypercarbia, hyperlactemia, loss of the adenosine pool, loss of ion gradients, and loss of water control.^5^, ^6^, ^7^, ^8^, ^9^

Our inability to connect our understanding of the metabolic injury caused by CA to the diagnosis and treatment of patients is a major impediment to progress in the field of resuscitation science. There are promising clinical treatments, such as therapeutic hypothermia, ischemic postconditioning, and controlled reperfusion, which improve survival in preclinical studies and are hypothesized to address postresuscitation metabolic disorders.^10^, ^11^, ^12^, ^13^ However, there are a number of randomized controlled clinical trials that show no benefit,^14^, ^15^, ^16^, ^17^, ^18^ in part as a result of the lack of an ability to differentiate patients with treatable metabolic injury, patients who might live with proper treatment, from patients who do not have a metabolic injury, patients who will live, or from patients with untreatable metabolic injury, patients who will not live.^4^, ^19^, ^20^, ^21^

Because of the central role of VO_2_ and CO_2_ production (VCO_2_) in mitochondrial energy metabolism, and the evidence that mitochondrial function is altered after resuscitation from CA,^22^, ^23^, ^24^ we hypothesize that the metabolic injury sustained by resuscitated animals will manifest as alterations in systemic VO_2_ and VCO_2_. While ex vivo results suggest that VO_2_ will decrease, we have purposefully left our hypothesis open because the conditions used in ex vivo experimentation do not reflect the postresuscitation cellular milieu. In order to test our hypothesis, we developed a novel system for the measurement of VO_2_ and VCO_2_ in intubated rats, and measured the VO_2_ and VCO_2_ to enable the calculation of the respiratory quotient (RQ) in a rat model of severe CA. VO_2_ and VCO_2_ were measured after return of spontaneous circulation (ROSC) at an inspired O_2_ concentration of 30% and were compared with the results from animals that received a sham surgery. In addition, we tested whether the supplementation of inspired 100% O_2_ gas affected the VO_2_, VCO_2_, or RQ in survivors of CA and compared results from animals receiving 100% O_2_ with animals receiving 30% O_2_ or the sham surgery. We tested the effect of supplemental O_2_ on aerobic respiration after CA to determine whether the toxic effects of supplemental O_2_ found in other injury models^25^ are replicated in this CA model.

## Methods

The Institutional Animal Care and Use Committees of the University of Pennsylvania, the Children's Hospital of Philadelphia, and the Feinstein Institute for Medical Research approved the study protocol. The data that support the findings of this study are available from the corresponding author upon reasonable request.

### Animal Preparation

We performed all instrumentation according to the previously described protocol.^26^ In brief, 34 adult male Sprague‐Dawley rats (450–550 g, Charles River Laboratories) were anesthetized with 4% isoflurane (Isosthesia, Butler‐Schein AHS) and intubated with a 14‐gauge plastic catheter (Surflo, Terumo Medical Corporation). We used male rats to avoid potential differences among animal subjects that may be caused by hormonal or genetic differences rather than differences from the experimental intervention (ie, to minimize potential sources of variability). Animals were mechanically ventilated (Ventilator Model 683, Harvard Apparatus) at a minute ventilation (MV) volume of 180 mL/min and a respiratory rate of 45 breaths per minute. In this study, we used 1 ventilation setting for all animals at all times and did not change the MV or respiratory rate over the experiments. All measured end‐tidal CO_2_ values were within a range of 35 to 45 mm Hg during preparation. Anesthesia was maintained with isoflurane 2% and fraction of inspired O_2_ (FIO_2_) 0.3. The left femoral artery was cannulated (sterile polyethylene‐50 catheter inserted for 20 mm) for continuous arterial pressure monitoring (MLT844, ADInstruments; Bridge Amplifier ML221, ADInstruments). A temperature probe (T‐type thermocouple probes, ADInstruments) was placed in the esophagus for continuous temperature monitoring. The core temperature was maintained at 36.5±0.5°C during the surgical procedure. The left femoral vein was cannulated with a polyethylene‐50 catheter, which was advanced into the inferior vena cava for drug infusion. This catheter was flushed with 150 U of heparin (Heparin, SAGENT Pharmaceuticals). At the end of the preparation, a blood sample (0.5 mL) was collected from the arterial catheter line and a blood gas analysis (i‐STAT, Heska) was performed.

### Experimental Protocol: CA and Sham Animals

**Figure 1:**
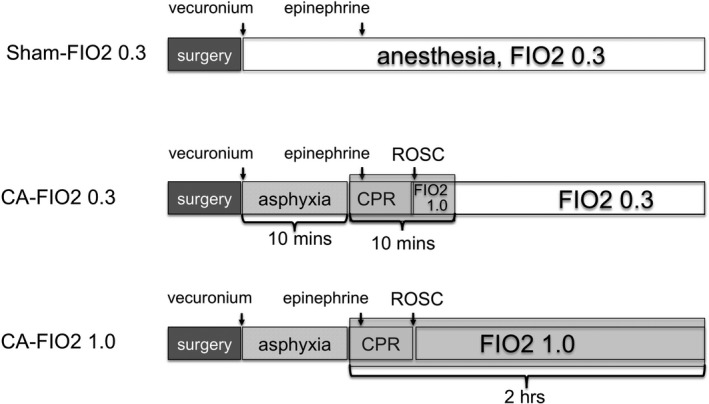
Study protocol. Animals were randomized into 3 groups at the end of surgery: (1) successful resuscitation from 10‐minute asphyxia arrest treated with inhaled 100% O_2_: cardiac arrest (CA)–fraction of inspired O_2_ (FIO_2_) 1.0 group (n=12); (2) successful resuscitation from 10‐minute asphyxia arrest treated with inhaled 30% O_2_: CA‐FIO_2_ 0.3 group (n=10); and (3) surgical sham control treated with inhaled 30% O_2_: sham‐FIO_2_ 0.3 group (n=12). CPR indicates cardiopulmonary resuscitation; ROSC, return of spontaneous circulation.

Animals were randomized into 3 groups at the end of surgery: (1) successful resuscitation from 10‐minute asphyxia arrest treated with inhaled 100% O_2_: CA‐FIO_2_ 1.0 group (n=12); (2) successful resuscitation from 10‐minute asphyxia arrest treated with inhaled 30% O_2_: CA‐FIO_2_ 0.3 group (n=10); and (3) surgical sham control treated with inhaled 30% O_2_: sham‐FIO_2_ 0.3 group (n=12) (Figure 1). After instrumentation, neuromuscular blockade was achieved by slow intravenous administration of 2 mg/kg of vecuronium bromide (Hospira) for all groups of animals. For animals in the CA groups, asphyxia was induced by switching off the ventilator and CA occurred 3 to 4 minutes after asphyxia started. We defined CA as a mean arterial pressure of <20 mm Hg; CA was completely untreated during this initial 10‐minute interval. After the initial 10 minutes, mechanical ventilation was restarted at an FIO_2_ of 1.0 and manual cardiopulmonary resuscitation (CPR) was delivered to all CA animals. Isoflurane inhalation was discontinued after the induction of asphyxia and was not given to CA animals after successful resuscitation. Chest compressions were performed with 2 fingers over the sternum at a rate of 260 to 300 per minute. Immediately after beginning CPR, a 20 μg/kg bolus of epinephrine was given to animals through the venous catheter. Following ROSC, defined as systolic blood pressure >60 mm Hg, CPR was discontinued. If ROSC did not occur by 5 minutes of CPR, resuscitation was terminated. Ten minutes after CPR started, FIO_2_ was switched back to 0.3 in the CA‐FIO_2_ 0.3 group, while it was kept at 1.0 in the CA‐FIO_2_ 1.0 group. We monitored animal physiology, VO_2_, and VCO_2_ for 120 minutes after resuscitation. For animals in the sham‐FIO_2_ 0.3 group, the same surgical procedures were performed, including vecuronium and epinephrine injections, without asphyxia or CPR. The animals were consistently anesthetized with 2% inhaled isoflurane/30% O_2_ during the entire series of measurements (Figure 1). All surgical procedures including resuscitation were performed by 1 investigator and therefore blinding procedures were not applied. Blood gas analysis was performed at 10, 20, 30, 45, 60, and 120 minutes after starting CPR. Mechanical ventilation was discontinued 120 minutes after CPR and animal survival was monitored up to 72 hours. Surviving animals were euthanized 72 hours after CPR. Postsurgical care including animal housing and observation were provided by facility in a blinded manner.

### Measurement Preparation

**Figure 2:**
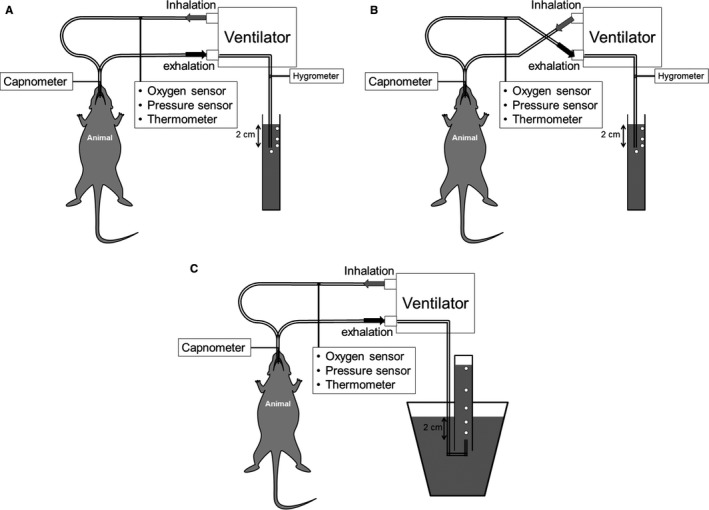
Illustration of the mechanical ventilation circuit with 2 sensors for measurements of O_2_ and CO_2_ concentrations. A, Before and after O_2_ consumption (VO_2_) measurements, fraction of inspired O_2_ (FIO_2_) is measured for at least 10 minutes. B, During measurements of VO_2_, fraction of expired O_2_ (FEO_2_) is measured after the ventilator circuit has been switched. O_2_ extraction is calculated by a subtraction between FIO_2_ and FEO_2_. C, Volume of exhaled gas (V_E_) was measured using a water bath and a 500 mL cylinder. For each measurement, expired gas was collected for 2 minutes and minute ventilation of V_E_ was calculated.

A photoluminescence‐quenching sensor (FOXY AL300 Oxygen Sensor Probe, Ocean Optics) and a fluorometer (NEOFOX‐GT, Ocean Optics) were used to measure the gas concentration of O_2_. O_2_ was measured inline in the exhalation or inhalation branch of the ventilator circuit (Figure 2A and 2B). A micro‐capnograph (Micro‐Capnograph CI240, Columbus Instruments) was used to measure CO_2_. CO_2_ was measured using a continuous side‐stream sample taken from the endotracheal tube of the intubated animal (Figure 2A and 2B). The sampling rate was set at 10 mL/min. The mechanical ventilation duty cycle was 50%, meaning that 5 mL/min was taken from both the inspiration and the expiration. Under a constant temperature condition, we manually calibrated the O_2_ and CO_2_ sensors before each experiment. Two‐point calibration was performed and medical air and medical O_2_ (General Welding Supply Corp.) were used for calibrating the O_2_ sensor. We used 20.9% as a low reference and 100.0% as a high reference. We used medical air (0% CO_2_) and industrial CO_2_ (10.4% CO_2_, General Welding Supply Corp.) for calibrating the CO_2_ sensor. Gas humidity was measured inline with a hygrometer (TFH 620, ebro). The calibration was completed at a humidity of 0%. A temperature probe (T‐type thermocouple probes, ADInstruments) and a pressure probe (MLT844, ADInstruments; Bridge Amplifier ML221, ADInstruments) were placed inline (Figure 2A and 2B). Ambient temperature and pressure were also monitored outside of the ventilator system (T‐type thermocouple probes, ADInstruments; Traceable Workstation Digital Barometer, Fisher Scientific).

### VO_2_ and VCO_2_ Measures in Intubated Rats

**Figure 3:**
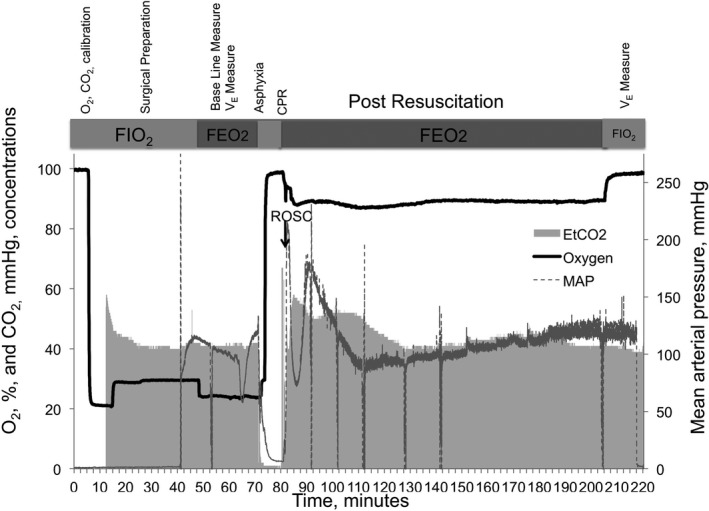
Experimental course of an animal. The figure depicts a typical experimental course of an animal in the cardiac arrest (CA)–fraction of inspired O_2_ (FIO_2_) 1.0 group. We firstly performed sensor calibrations for each experiment. Animal preparation was completed within 40 minutes after successful intubation. Baseline FIO_2_ was measured during surgical preparation. Fraction of expired O_2_ (FEO_2_) was then measured by swapping the in/out ports in the ventilator circuit. We measured FEO_2_ and monitored animal physiology, O_2_ consumption (VO_2_), and CO_2_ production (VCO_2_) for 2 hours after resuscitation. FIO_2_ was measured twice in each VO_2_ measurement, and before and after a 2‐hour period of FEO_2_ measurement. CPR indicates cardiopulmonary resuscitation.

The fluorometer measures O_2_ concentration every 100 ms. The fluorometer performed a windowed average of these measurements, and exported a value every second. The time constant for the fluorometer is about 300 ms. Given the ventilation rate, this sensor is too slow to perform mainstream O_2_ concentration measurements in the endotracheal tube. For this reason, the O_2_ sensor was placed in the middle of a ventilator tubing arm ≈100 cm from the endotracheal tube (Figure 2). O_2_ concentrations were measured in the inspiratory arm (FIO_2_) or in the fraction of expired O_2_ arm (FEO_2_) by swapping which ventilator circuit arm was connected to the in and out port of the ventilator. Given enough equilibration time after switching ventilator port connections, this setup provided relatively constant O_2_ concentrations for measurement. The differences between the alveolar and tracheal O_2_ concentrations lead to a concentration gradient in the expiratory arm of the ventilator circuit. However, the relatively large volume of our ventilator circuit (≈15 mL) compared with our tidal volume (≈4 mL) allowed sufficient time for gas diffusion, which significantly decreased the O_2_ concentration gradients in the expiratory arm of the circuit. After the experiments, the exported O_2_ concentrations were averaged every minute, and measurements made in the inspiratory arm are reported as FIO_2_ and measurements made in the expiratory arm are reported as FEO_2_. Figure 3 depicts a typical experimental course of an animal in the CA‐FIO_2_ 1.0 group. Sensors were calibrated before each experiment. Animal preparation was completed within 40 minutes after successful intubation. Baseline FIO_2_ was measured during surgical preparation. FEO_2_ was then measured by swapping the in/out ports in the ventilator circuit (Figure 2A and 2B). FIO_2_ was measured twice in each VO_2_ measurement for a period of at least 10 minutes, before and after the 2‐hour period of FEO_2_ measurement. From asphyxia to starting CPR, the rats were disconnected from the ventilator circuit and so a test lung (small rubber balloon) was connected to the circuit. FIO_2_ was measured with the test lung while the animal was in asphyxia. After 10 minutes of asphyxia, the animal was reconnected to the ventilator circuit, the ventilator circuit was adjusted to measure FEO_2_, and CPR was initiated.

**Figure 4:**
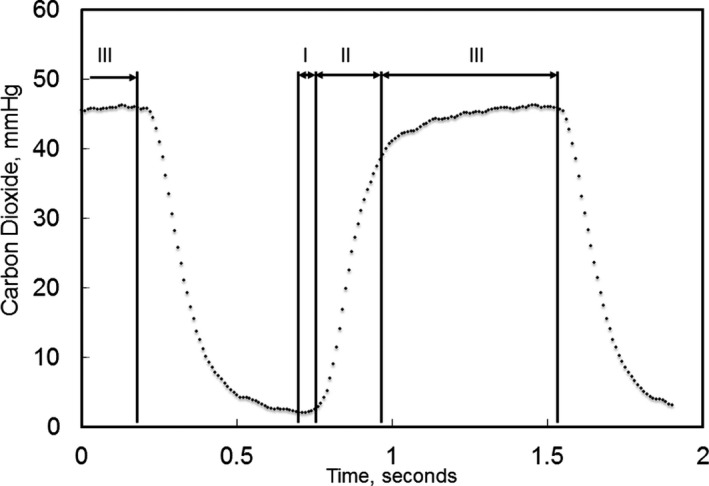
Volumetric capnograph. Phase Ι is a starting point of an expiration with a tiny elevation of the curve of partial pressure of carbon dioxide (PCO_2_) followed by a steep slope of phase ΙΙ. The end of expiration at phase ΙΙΙ is defined as end‐tidal PCO_2_.

Because we measured CO_2_ concentration using a side‐stream breath‐by‐breath method, we used a slightly different calculation for fractional concentration of CO_2_ in exhaled gas (FECO_2_). To estimate FECO_2_, we quantitated % concentration of CO_2_ from the capnograph waveform (Figure 4).^27^ The small increase in partial pressure of carbon dioxide (PCO_2_), noted as phase Ι in Figure 4, marks the transition from inspiration to expiration. This is followed by a significant increase in PCO_2_, labeled phase ΙΙ. The end of expiration at phase ΙΙΙ is defined as end‐tidal PCO_2_. Capnograph data were extracted every 10 ms. We calculated the first derivative of the capnograph to detect the beginning of phase I and the end of phase III. The interval starting from the beginning of phase I to the end of phase III is considered the expiratory phase of the ventilation. Using this method, we segmented the capnograph into inspiratory and expiratory phases. Using only the expiratory phases of the capnograph, we averaged 1 minute worth of FECO_2_ measurements. The CO_2_ sensor is affected by changes in the O_2_ concentration in the sampled gas. Therefore, we corrected CO_2_ measurements empirically based on our sensor calibration experiments, described below.

**Figure 5:**
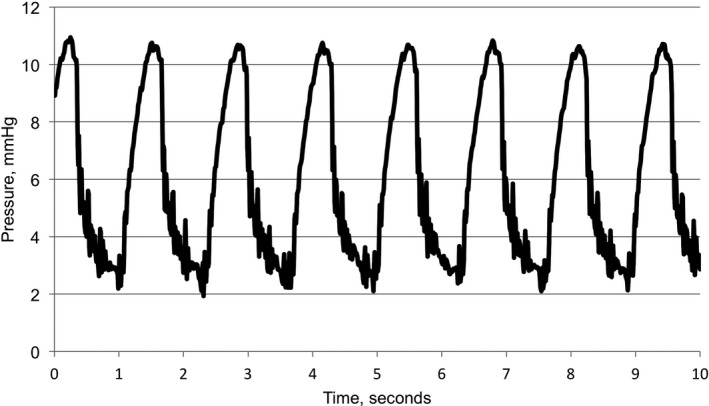
Pressure curve inside the mechanical ventilator circuit. Ventilator airway pressure was measured through our experiment so we could verify that the pressure inside was kept positive by a positive end‐expiratory pressure of 2 cm.

To measure volume of exhaled gas (V_E_), we used an inverted, water‐sealed 500 mL cylinder that was filled with water. Exhaled gas displaced the water in the cylinder during volume measurements. Exhaled gas was collected for 2 minutes and the MV volume was calculated. The resolution of the measurement was 2.5 mL/min. V_E_ measurements were performed twice in each experiment before and after a 2‐hour period of metabolic measurement (Figure 2C). Ventilator airway pressure was measured through our experiment so we could verify that the pressure inside was kept positive by a positive end‐expiratory pressure of 2 cm (Figure 5). The positive pressure inside the circuits ensures that the circuit is not contaminated by ambient air. We regularly inspected the rat oropharynx visually to eliminate the possibility of a major leak around the endotracheal tube. Since the airway pressure was positive, gas leak through the space between the endotracheal tube and the vocal cords would create bubbles in the oropharynx. In addition, we compared the V_E_ measurements before and after the metabolic measurement. The comparison of post‐V_E_ to pre‐V_E_ was made for each experiment and the average ratio of post‐V_E_/pre‐V_E_ was 1.011 (95% confidence interval, 0.994–1.028 [n=34]). This shows that the CA injury model had no observable effect on our V_E_ measurements.

### Gas Concentration Measurements: Temperature Control

Gas concentration measurements are temperature dependent. The O_2_ and CO_2_ measurements used different temperature control techniques. The micro‐capnograph uses a warming chamber to warm the sampled gas to 40°C. We used a water bath and copper tubing to control the temperature for the O_2_ sensor. The fluorometer simultaneously measures fluorescence and sample temperature in order to correct for temperature effects. The water bath was kept in a narrow temperature range of 19 to 21°C.

### Measurement Validation

Four different 4% CO_2_ gas mixtures (4% CO_2_ contained in 10%, 45%, 80%, and 96% O_2_; MESA International Technologies, Inc) were prepared. The accuracy of O_2_ and CO_2_ measurements was tested using these mixtures. The error of our O_2_ measurements was ±0.7% at a range from 20.9% to 100%. Because we swapped the in/out ports and used 1 sensor for measuring FIO_2_ and FEO_2_, the error (uncertainty) of FIO_2_ and FEO_2_ were dependent. Since the propagation of uncertainty for subtraction of FIO_2_−FEO_2_ keeps the covariance, σFIO_2_·FEO_2_, in its calculation the error of O_2_ extraction rate, FIO_2_−FEO_2_, is calculated as ±0.7% at a range from 20.9% to 100%.

**Figure 6:**
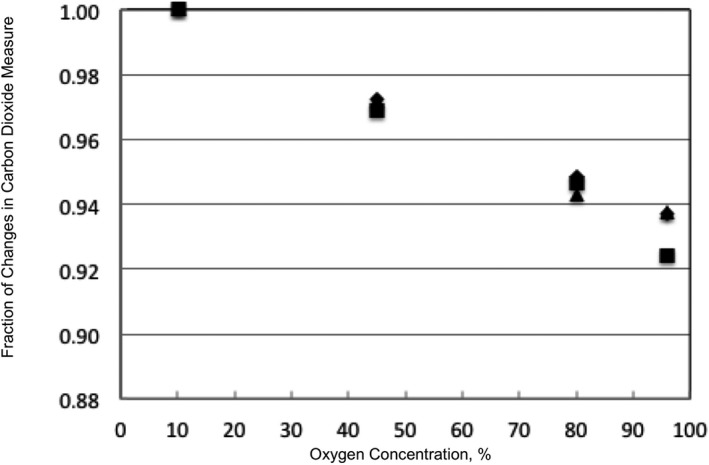
Effect of O_2_ concentration on the CO_2_ sensor. Four different mixture gases (O_2_ 10% and CO_2_ 4%, O_2_ 45% and CO_2_ 4%, O_2_ 80% and CO_2_ 4%, and O_2_ 96% and CO_2_ 4%) were used. The experiment was performed under humid conditions at a relative humidity of 92.8±3.5% at a gas temperature of 21.3±0.6°C.

We tested the effect of different O_2_ concentrations on our CO_2_ sensor under humid conditions. Deionized pure water was poured into Tygon tubing (Saint‐Gobain) and it was warmed to 50°C by a hot water bath. The gas mixture was passed through the tubing and the CO_2_ concentration was measured for each mixture. In order to reduce CO_2_ measurement errors caused by changes in O_2_ concentrations, we measured the effect of different O_2_ concentrations on the CO_2_ sensor and created an equation to correct the CO_2_ errors, as shown in Figure 6. The CO_2_ concentration measurement decreased linearly as the O_2_ concentration increased. Humidity was 92.8±3.5% at a gas temperature of 21.3±0.6°C. Based on this result, we derived the following empirical equation:

(1) $$CO_{2}=\frac{\text{mC}O_{2}}{-0.00077\times O_{2}+1.0077}$$

where, CO_2_ is the estimated concentration, mCO_2_ is the measured value, O_2_ is the concentration of O_2_ (%).

Temperature and pressure are the major components that affect the volume measure of a gas. Therefore, we tested whether there were differences in temperature and/or pressure between inhaled and exhaled gases before and after swapping the in/out ports. Data were obtained 1 minute before and after swapping the in/out ports of the ventilator circuit. As can be seen in Table 1, the gas temperature was 19.69±0.46°C when FIO_2_ was measured and it increased by 0.01°C when FEO_2_ was measured. Even though exhaled gas from animals is warmer than inhaled gas supplied from a mechanical ventilator, it did not make a significant impact on our measurements because the water bath system controls the temperature. The pressure inside the circuit was 5.62±0.42 mm Hg when FIO_2_ was measured and it decreased by 0.19 mm Hg when FEO_2_ was measured.

**Table 1:** Gas Conditions for FIO_2_ and FEO_2_ Measurements of Experimental Animals

	FIO_2_ (n=5)	FEO_2_ (n=5)	*P* Value
Gas temperature inside ventilator circuit, °C	19.69±0.46	19.70±0.46	<0.01
Gas pressure inside ventilator circuit, mm Hg	5.62±0.42	5.43±0.44	<0.01
Ambient temperature, °C	21.69±0.26	21.67±0.15	0.886

Values are expressed as mean±SD. The data were collected 1 minute before and after swapping the in/out ports in the ventilator circuit. The results are shown as the average and SD of data obtained from 5 animals. Paired‐sample *t* test was used to compare the values between fraction of inspired O_2_ (FIO_2_) and fraction of expired O_2_ (FEO_2_).

The estimation of inspiration volume by the Haldane transformation^28^ is limited to FIO_2_ <0.6.^29^ We needed a method that was applicable to FIO_2_ of 1.0. Therefore, we performed an experiment to measure the effect of CO_2_ production and O_2_ consumption on the difference between V_I_ and V_E_, where V_I_ and V_E_ are MV volumes of the mechanical ventilation in the inhaled and exhaled gases, respectively. Before conducting the experiment, the ventilator and ventilator circuit were leak tested. The first part of the experiment was the determination of V_I_. The volume of gas exiting the ventilator per minute was measured in the presence of a test lung. Because the system was leak tested, and there was no gas exchange in the test lung, the volume of gas exiting the ventilator is equivalent to the volume of gas entering the test lung, which is V_I_. This measurement was made in triplicate. Then, an animal was anesthetized and intubated. The side‐stream capnography measurement was omitted and the port sealed so that no ventilation volume was lost. The volume of gas exiting the ventilator was then measured in triplicate. In this setting, the measured volume is V_E_. Next, the animal was euthanized by stopping mechanical ventilation under a surgical plane of anesthesia and neuromuscular blockade. This resulted in asphyxia and CA. V_E_ was measured in triplicate 15 and 45 minutes after euthanasia. Because the animal was euthanized, the ventilations also resulted in no gas exchange with the animal. The results of these experiments are reported in Table 2. V_I_ measured using the rubber‐test lung was 177.5 mL/min. As can be seen in the Table 2, there were no detectable differences between V_E_ and V_I_ in the animal. The data support discarding the Haldane transformation in favor of the assumption that V_I_ equals V_E_ in animals, particularly for high FIO_2_ values. However, it is possible that the difference between V_I_ and V_E_ is smaller than the resolution of our volume measurement. The resolution of our volume measurement was 2.5 mL/min and so it was ≈1.4% of an MV of 180 mL/min. This number indicates that V_I_ can be 1.4% more or less than V_E_.

**Table 2:** Metabolic Effect on V_E_ of an Animal

Test Lung V_I_=V_E_, mL/min	Animal—Alive V_E_, mL/min	Animal—Dead	
		15 min V_I_=V_E_, mL/min	45 min V_I_=V_E_, mL/min
177.5±0	177.5±0	176.7±1.4	177.5±0

One animal was used in this experiment. The volume measurements were repeated 3 times at each condition. Values are expressed as mean±SD. V_E_ indicates volume of exhaled gas; V_I_, volume of inspired gas.

### Calculations

VO_2_ and VCO_2_ are calculated by the following equations^29^, ^30^:

(2) $$VO_{2}=V_{I}\times \text{FI}O_{2}-V_{E}\times \text{FE}O_{2}$$

(3) $${\text{VCO}}_{2}=V_{I}\times {\text{FICO}}_{2}-V_{E}\times {\text{FECO}}_{2}$$

In our calculation, FICO_2_ is assumed to be zero since the inhaled gas did not contain CO_2_. It was not appropriate to use the Haldane transformation^28^ to estimate V_I_ from V_E_ because we used inhaled O_2_ concentrations of 100% in the CA‐FIO_2_ 1.0 group.^29^ Based on our validation study, we determined that it was reasonable to assume that V_I_ equals V_E_ for this experimental setup; this assumption results in the following calculation of VO_2_:

(4) $${\text{VO}}_{2}=V_{E}\times \left({\text{FIO}}_{2}-{\text{FEO}}_{2}\right)$$

Room temperature and atmospheric pressure were measured in each experiment so that VO_2_ and VCO_2_ could be calculated at standard temperature and pressure. The RQ was calculated by the following equation:

(5) $$\text{RQ}={\text{VCO}}_{2}/{\text{VO}}_{2}$$

(6) $$\text{RQ}=\frac{{\text{FECO}}_{2}}{{\text{FIO}}_{2}-{\text{FEO}}_{2}}$$

Different FIO_2_ settings affect errors in VO_2_ caused by the assumption that V_I_ equals V_E_. In order to evaluate errors in VO_2_ of our study, we calculated %error from the following equation:

(7) $$\%\text{error}=\frac{\text{Exact}-\text{Approximate}}{\text{Approximate}}\times 100$$

Exact VO_2_ was calculated by equation (2), approximate VO_2_ was calculated by equation (4), and equation (6) is transformed to:

(8) $$\%\text{error}=\frac{\left(\text{VI}\times {\text{FIO}}_{2}-\text{VE}\times {\text{FEO}}_{2}\right)-\text{VE}\times \left({\text{FIO}}_{2}-{\text{FEO}}_{2}\right)}{\text{VE}\times \left({\text{FIO}}_{2}-{\text{FEO}}_{2}\right)}\times 100$$

(9) $$\%\text{error}=\left(\frac{\text{VI}}{\text{VE}}-1\right)\times \left(\frac{{\text{FIO}}_{2}}{{\text{FIO}}_{2}-{\text{FEO}}_{2}}\right)\times 100$$

The resolution of our V_E_ measurement was 2.5 mL/min and it was 1.4% of the MV volume (180 mL/min). We assumed that V_I_ equals V_E_ but exact V_I_ might be either more or less than V_E_ (±1.4%). Therefore, the %error of our measurements is:

(10) $$\%\text{error}=\pm 1.4\times \left(\frac{{\text{FIO}}_{2}}{{\text{FIO}}_{2}-{\text{FEO}}_{2}}\right)$$

The %error becomes greater when higher inhaled O_2_ is used and it becomes smaller when the animal's O_2_ extraction rate, FIO_2_−FEO_2_, is high. In our injured animal model, the average of O_2_ extraction rate was 7.5% when inhaled O_2_ was 30%, where it was 9.3% when inhaled O_2_ was 100%. Therefore, the %error of our VO_2_ measurements was estimated as ±5.6% in the CA‐FIO_2_ 0.3 group and ±15.1% in the CA‐FIO_2_ 1.0 group.

### Statistical Analysis

We reported continuous data as mean and SD. Group comparisons were made with ANOVA, and Scheffé multiple comparison procedure was used for post hoc analyses. The numbers of VO_2_, VCO_2_, and RQ were compared within the 3 different test groups (CA‐FIO_2_ 1.0 group, CA‐FIO_2_ 0.3 group, and sham‐FIO_2_ 0.3 group) at each time point. We used Fisher exact test for comparison of categorical outcomes and log‐rank test for comparison of survival curves among groups. A 2‐tailed *P*<0.05 was considered statistically significant. All calculations were performed with SPSS Statistics version 22 for Mac (IBM).

## Results

### CA Animal Models

**Figure 7:**
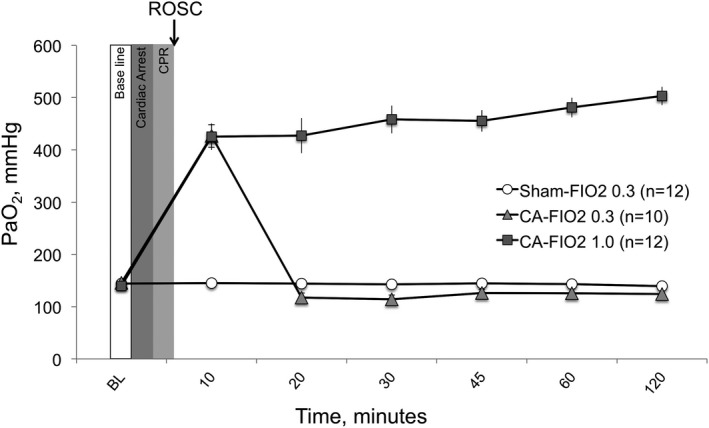
Arterial O_2_ pressure (arterial partial pressure of O_2_ [PaO_2_], mm Hg) among the 3 groups. Values are expressed as mean±SE. CA indicates cardiac arrest; CPR, cardiopulmonary resuscitation; FIO_2_, fraction of inspired O_2_; ROSC, return of spontaneous circulation.

**Figure 8:**
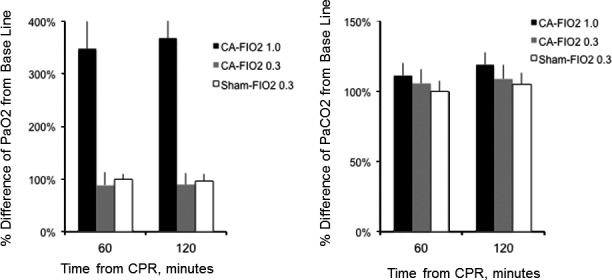
Arterial partial pressure of oxygen (PaO_2_) and arterial partial pressure of carbon dioxide (PaCO_2_) differences from baseline. The %difference was compared from baseline in a value of PaO_2_ at 60 minutes after cardiopulmonary resuscitation (CPR) and 120 minutes after CPR. The %difference was compared from baseline in a value of PaCO2 at 60 minutes after CPR and 120 minutes after CPR. There were no statistical differences at any time points.

**Figure 9:**
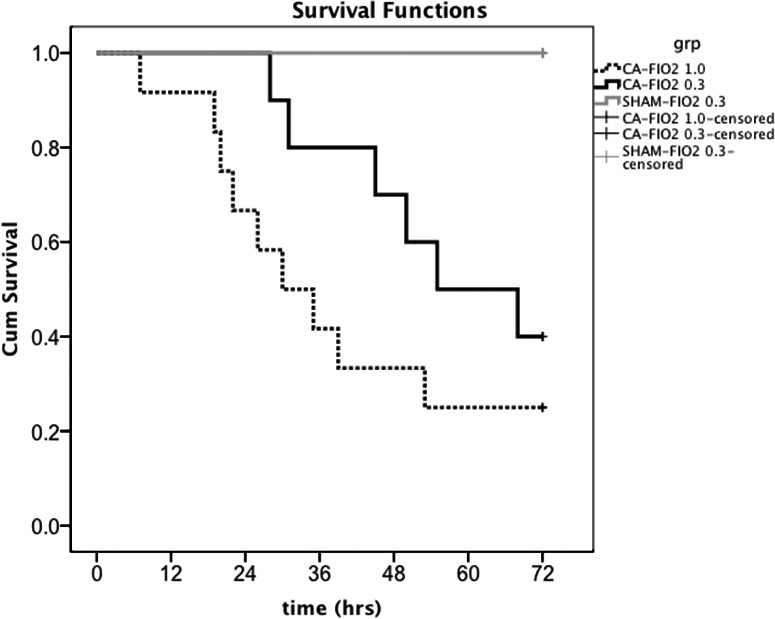
Kaplan–Meier survival curves among the 3 groups. Log‐rank test shows no significant differences between the cardiac arrest (CA)–fraction of inspired O_2_ (FIO_2_) 1.0 and CA‐FIO_2_ 0.3 groups (*P*=0.164).

No significant differences in basal characteristics were found between the groups (Table 3). Arterial O_2_ pressures for the 3 groups are shown as a function of time in Figure 7. As previously mentioned, we used 1 ventilation setting (respiratory rate: 45 breathes per minute; MV: 180 mL/min) for all animals at all times and did not change the MV or respiratory rate over the experiments. All measured end‐tidal CO_2_ values were within a range of 35 to 45 mm Hg during surgical preparation. Mean PaO_2_ remained significantly higher in the CA‐FIO_2_ 1.0 group compared with the CA‐FIO_2_ 0.3 and the sham‐FIO_2_ 0.3 groups. The %difference of PaO_2_ from baseline decreased by 11% in the CA‐FIO_2_ 0.3 group at 60 and 120 minutes from CPR. There were no statistical differences between the CA‐FIO_2_ 0.3 group and the sham‐FIO_2_ 0.3 group (Figure 8). The %difference of arterial partial pressure of carbon dioxide from baseline increased by 11% at 60 minutes and by 19% at 120 minutes in the CA‐FIO_2_ 1.0 group, and increased by 5% at 60 minutes and by 9% at 120 minutes in the CA‐FIO_2_ 0.3 group. There were no statistical differences between the 3 groups at either time point (Figure 8). Seventy‐two‐hour survival curves for the 3 groups are shown in Figure 9. Survival rates in the CA‐FIO_2_ 1.0 and the CA‐FIO_2_ 0.3 groups were not found to be different (*P*=0.164) using log‐rank test. Using Fisher exact test, the survival rates of CA‐FIO_2_ 1.0 versus CA‐FIO_2_ 0.3 at each time point (24, 36, 48, and 72 hours after CA) were 67% (8/12) versus 100% (10/10, *P*=0.096), 42% (5/12) versus 80% (8/10, *P*=0.099), 33% (4/12) versus 70% (7/10, *P*=0.198), and 25% (3/12) versus 40% (4/10, *P*=0.652), respectively. No animal died in the sham‐FIO_2_ 0.3 group.

**Table 3:** Basal Characteristics and Resuscitation Data in the 3 Groups

	CA‐FIO_2_ 1.0 (n=12)	CA‐FIO_2_ 0.3 (n=10)	Sham (n=12)
Weight, g	479±32	462±12	480±36
CPR to ROSC time, s	109±37	100±30	···
Tidal volume, mL/kg	7.4±0.6	7.7±0.2	7.2±0.8
FIO_2_	0.31±0.02	0.31±0.01	0.31±0.01
Body temperature, °C	36.6±0.3	36.7±0.4	36.9±0.4
Blood gas analysis			
pH	7.46±0.03	7.44±0.02	7.44±0.02
PO_2_, mm Hg	140±17	146±16	144±18
PCO_2_, mm Hg	40±2	40±4	42±5
Lactate, mmol/L	1.3±0.4	1.3±0.4	1.1±0.5

Blood samples were obtained from arterial line before asphyxia (baseline). Return of spontaneous circulation (ROSC) time was defined as systolic blood pressure >60 mm Hg. Values are expressed as mean±SD. CA indicates cardiac arrest; CPR, cardiopulmonary resuscitation; FIO_2_, fraction of inspired O_2_; PCO_2_, partial pressure of carbon dioxide; PO_2_, partial pressure of O_2_.

### Alterations of VCO_2_, VO_2_, and RQ After CA

**Figure 10:**
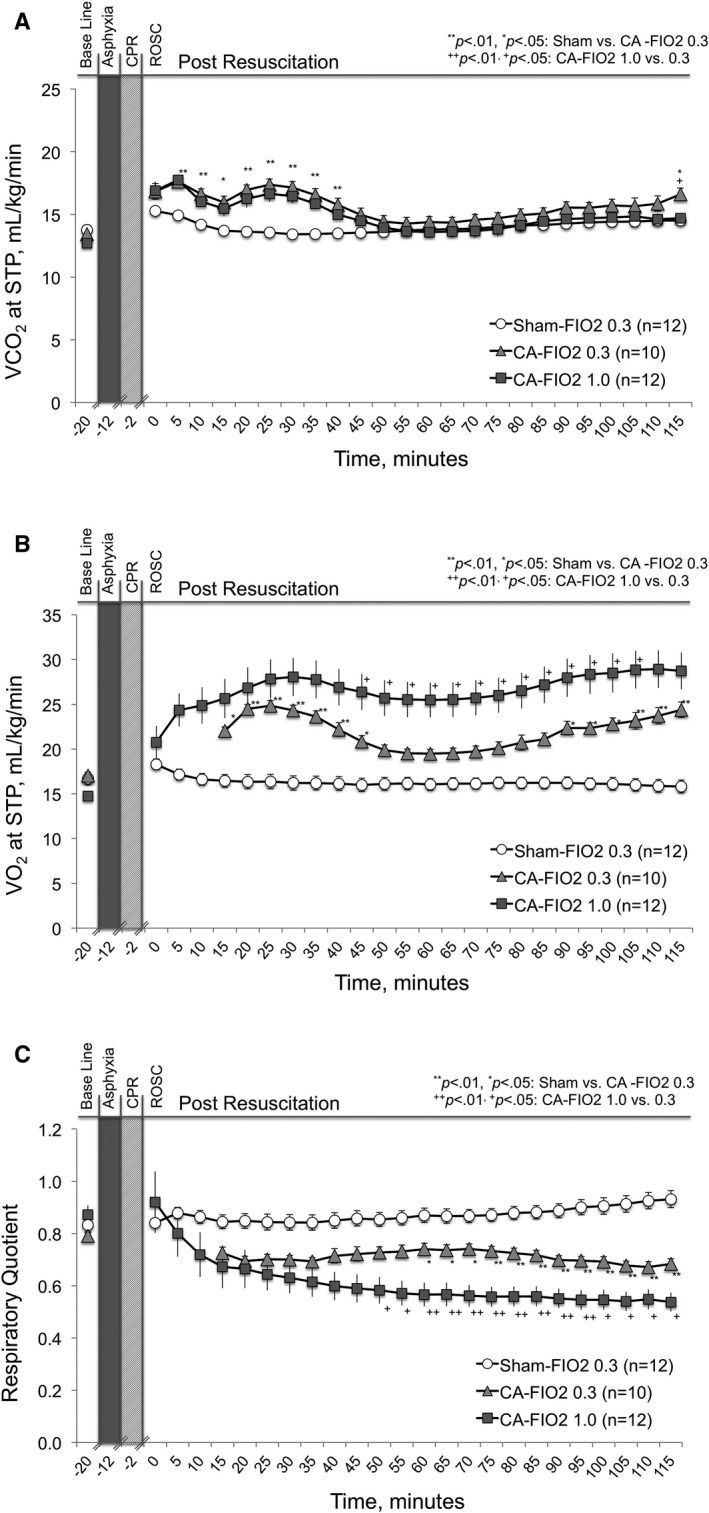
Alterations of CO_2_ production (VCO_2_), O_2_ consumption (VO_2_), and RQ after cardiac arrest (CA). A, VCO_2_ over time in the CA groups compared with the sham group; (B) VO_2_ over time in the CA groups compared with the sham group; and (C) respiratory quotient (RQ) over time in the CA groups compared with the sham group. CPR indicates cardiopulmonary resuscitation; ROSC, return of spontaneous circulation. Values are expressed as mean±SE. The numbers of VO_2_, VCO_2_, and RQ were compared within the 3 different test groups (CA–fraction of inspired O_2_ [FIO_2_] 1.0 group, CA‐FIO_2_ 0.3 group, and sham‐FIO_2_ 0.3 group) at each time point. Significant differences between the groups with a *P*<0.05 is indicated as *, *P*<0.01 ** (sham‐FIO_2_ 0.3 vs CA‐FIO_2_ 0.3), *P*<0.05 +, and *P*<0.01 ++ (CA‐FIO_2_ 0.3 vs CA‐FIO_2_ 1.0).

VCO_2_ was significantly greater in both CA groups compared with the sham group 25 minutes after ROSC (CA‐FIO_2_ 1.0: 16.7±2.2, *P*<0.01; CA‐FIO_2_ 0.3: 17.4±1.4, *P*<0.01; versus sham‐FIO_2_ 0.3: 13.6±1.1 mL/kg per minute at standard temperature and pressure, respectively). VCO_2_ in the CA groups returned to normal 45 minutes after ROSC (Figure 10A). As can be seen in Figure 10B, the VO_2_ value was greater in the CA groups than in the sham group. Values measured 115 to 120 minutes after ROSC were significantly different (CA‐FIO_2_ 1.0: 28.7±6.7, *P*<0.01; CA‐FIO_2_ 0.3: 24.4±2.3, *P*<0.01; versus sham‐FIO_2_ 0.3: 15.8±2.4 mL/kg per minute at standard temperature and pressure). As a result, the RQ was significantly and persistently lower in the CA groups than in the sham group over the 2‐hour measurement period (CA‐FIO_2_ 1.0: 0.54±0.12 versus CA‐FIO_2_ 0.3: 0.68±0.05 versus sham‐FIO_2_ 0.3: 0.93±0.11 in minutes 115 to 120 of ROSC, *P*<0.01 [Figure 10C]). The proportion (numbers) of animals that had an RQ <0.7 at the minimum was: 92% (11/12) in the CA‐FIO_2_ 1.0 group, 80% (8/10) in the CA‐FIO_2_ 0.3 group, and 0% (0/12) in the sham‐FIO_2_ 0.3 group.

In our CA injury model, the VO_2_ value increased by 50% when the FIO_2_ was 0.3. When the FIO_2_ value was 1.0, the VO_2_ increased by 80%.

## Discussion

The experiments reported here demonstrate that animals resuscitated from CA have reduced RQs, increased VO_2_ values, and similar VCO_2_ values, relative to their pre‐arrest values. The amount of O_2_ consumed in the post‐arrest period was dependent on the inhaled O_2_ concentration. These results confirm our contention that resuscitation from CA causes significant metabolic injury to the animal. What was not expected, and what has previously been considered impossible, is that rats resuscitated from CA could exhibit RQs significantly lower than 0.7. Our expectations had been that resuscitated animals would exhibit classical hypermetabolism or hypometabolism, eg, their RQ would remain unchanged, but they would exhibit either increased or decreased metabolic rate.

This study substantially improves our understanding of the use of indirect calorimetry as a real‐time metabolic monitor to track injury severity after resuscitation. One of our most important findings is that a resuscitated rat can sustain RQs outside of the commonly cited range of 0.7 to 1.0. RQ values <0.7 are often considered to be in error.^30^, ^31^, ^32^, ^33^ Oshima et al^32^ reported 7 cases of CA in which energy expenditures were found to be unexpectedly high in the post‐ROSC period, but excluded measurements when the RQ was lower than 0.7. Black et al^34^ reported the results of a careful study of mechanically ventilated patients in the intensive care unit with VO_2_ measured using 3 distinct methods. In that article, nearly 25% of collected data were excluded from analysis because the measured RQ was <0.6 or >1.2. An increase in the metabolic rate, eg, hypermetabolism, has been reported in patients with burns,^35^, ^36^ sepsis,^37^ and other diseases.^38^, ^39^ However, these reports did not include measurements of VCO_2_ and therefore it is possible that a shift in O_2_ metabolism, such as the one described here, was interpreted as hypermetabolism. In all, our results suggest that our finding of an RQ <0.7 may be more prevalent than previously suspected. Furthermore, our finding that changes in VO_2_ are not always linked to changes in VCO_2_ clarifies that claims of increased or decreased metabolic rate cannot be supported without measuring both VO_2_ and VCO_2_.

There are some studies that report VO_2_ after CA using the Fick method in rats,^40^, ^41^ but the reported post‐CA VO_2_ was either comparable to or lower than the baseline measurements or reference values in those studies. However, none of these studies measured VCO_2_, thus the RQ values remain unknown. Hensel and Kox^42^ reported that the number of VO_2_ measured by indirect calorimetry was higher than that by the Fick method in patients with lung infection. It was inferred that the injured lung itself might have measurable VO_2_.

**Figure 11:**
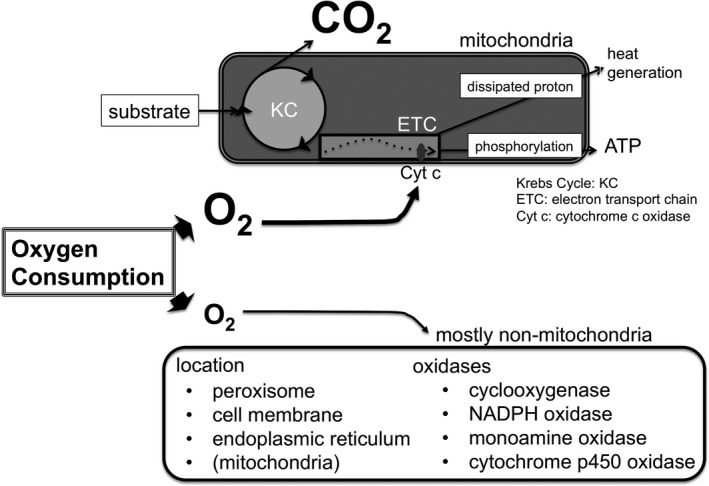
Pathways of cellular energy metabolism and O_2_ consumption (VO_2_). ATP indicates adenosine triphosphate; Cyt c, cytochrome c oxidase; ETC, electron transport chain; KC, Krebs cycle; NADPH, nicotinamide adenine dinucleotide phosphate.

We currently lack a biochemical explanation for the change in O_2_ utilization manifested in these experiments. Under normal aerobic respiration, nearly 95% of O_2_ is consumed as the terminal electron accepter within the mitochondria's electron transport chain (Figure 11).^43^, ^44^, ^45^ The electrons that are used to reduce O_2_ to water are supplied by the Krebs cycle. The mitochondrial oxidation of glucose consumes 6 O_2_ molecules for each 6‐carbon glucose molecule and generates 6 CO_2_ molecules to yield an RQ of 1, while the typical β‐oxidation of a fatty acid consumes 23 molecules of O_2_ for each 16‐carbon fatty acid, which will generate 16 CO_2_ molecules, yielding an RQ of 0.7. This stoichiometry defines the “normal” range for RQ. In our experiments, the production of CO_2_, and therefore the production of reducing equivalents by the Krebs cycle, returned to baseline after 45 minutes, but our VO_2_ increased to 150 or 200% of baseline, depending on the inspired O_2_ fraction. This change represents a fundamental shift in the cellular utilization of O_2_.

There is an ongoing debate about the balance between the potential benefits and dangers of supplemental O_2_. It is standard clinical practice to provide supplemental O_2_ when the O_2_ saturation is low.^38^ However, it has been shown that supplemental O_2_ increases mortality in some critical illness injury models.^25^ In our CA injury model, the VO_2_ increased by 50% when the FIO_2_ was 0.3. When the FIO_2_ was 1.0, the VO_2_ increased by 80%. Previous studies have shown that VO_2_ is not dependent on the FIO_2_ in healthy animals and humans.^28^, ^46^ This is consistent with our biochemical understanding of mitochondrial respiration, which suggests that the electron transport chain is nicotinamide adenine dinucleotide limited.^43^, ^47^ Therefore, we interpret our finding that VO_2_ is dependent on the inspired O_2_ fraction in the CA animal model as an indication that resuscitation may lead to metabolic processes or states that are O_2_ limited. In our injury model, the FIO_2_ 1.0 group trended toward higher mortality than the FIO_2_ 0.3 group. While this was not statistically significant with our numbers, it appears consistent with previously published results.^25^

The idea that resuscitation from CA results in “metabolic injury” has become resuscitation science dogma. Alterations in mitochondrial function have been detected in a variety of CA models in a variety of animal species.^22^, ^24^, ^48^ Postischemic conditioning has been shown to improve survival in swine resuscitated from CA.^49^ The American Heart Association's guidelines highlight reperfusion injury as a problem resuscitated patients will face.^19^, ^50^ While it remains unclear how our findings relate to this spectrum of metabolic injuries, we have demonstrated that significant differences in the metabolisms of resuscitated rats can be detected using indirect calorimetry. Interestingly, the global metabolic rate in the resuscitated rat did not appear to be increased or decreased. Instead, it appears that additional O_2_ consuming processes are making a significant contribution to the total VO_2_ of the animal.

We believe this novel metabolic phenotype in rats resuscitated from prolonged CA is in vivo evidence of postresuscitation metabolic injury, and might be used to estimate severity of postresuscitation injury. Measuring VO_2_, VCO_2_, and RQ is noninvasive and can be translated into larger animal and human studies. However, currently available medical devices are associated with measurement limitations that undermine their accuracy.^44^, ^51^, ^52^ However, a reliable device could be easily designed and constructed using existing technology, and we anticipate validating our finding in human survivors of CA.

There are no commercially available methods to measure VO_2_ and VCO_2_ in mechanically ventilated rodents. However, we have confidence in our O_2_ and CO_2_ measurements because our baseline VO_2_ and VCO_2_ values are consistent with reports from other rodent laboratories. For example, Barrow^36^ reported VO_2_ in the range of 13 to 18 mL/kg per minute (800–1100 mL/kg per hour) in rats with sham surgery, which matches our measured VO_2_ range of 16 to 18 mL/kg per hour in our sham group animals. Yamaoka et al^53^ used a commercially available metabolic chamber for rats and reported that VCO_2_ was 14 to 20 mL/min per kg (10–14 mL/min per kg0.75) at sleep and 25 to 31 mL/min per kg (18–22 mL/min per kg0.75) at normal activity. Musch et al^54^ reported that VCO_2_ was 20 mL/min per kg at rest and 67 to 72 mL/min per kg at maximal exercise. VCO_2_ in our sham group animals was 14 to 16 mL/min per kg. This number is close to that of sleeping animals but less than animals at normal activity or rest. VCO_2_ of the baseline and the sham surgery animals were measured under anesthesia and it is reasonable that the number we reported was close or less than those of nonanesthetized animals. We interpret this congruence with reported values as evidence that our system provides reasonable measurements.

There are at least 3 biochemical pathways that could explain the imbalance between VO_2_ and VCO_2_. The first is that cytochrome c oxidase is consuming the additional O_2_ with extra reducing equivalents being provided by processes that do not generate CO_2_, eg, processes other than the Krebs cycle such as glycerol‐phosphate shuttle and/or the malate‐aspartate shuttle pathways providing the electron transport chain with FADH_2_ and nicotinamide adenine dinucleotide, respectively.^55^, ^56^ The second is that the amount of O_2_ being consumed by oxidases and oxygenases other than cytochrome c oxidase has increased. Nonmitochondrial VO_2_ has been described (Figure 11)^57^, ^58^ and these reactions do not involve nicotinamide adenine dinucleotide or FADH_2_ in mitochondria, and therefore are not coupled to CO_2_ created by the Krebs cycle. Enzymes such as nicotinamide adenine dinucleotide phosphate oxidases, cytochrome p450 oxidases, monoamine oxidases, and xanthine oxidases^59^ primarily oxidize water without a concomitant production of CO_2_. The third is the prolonged production of reactive O_2_ species within the electron transport chain enzymes. The creation of superoxide requires a single electron to reduce molecular O_2_, where normal electron transfer requires 4 electrons to reduce molecular O_2_.^60^ This would shift the balance of VO_2_ and VCO_2_ in the direction we have observed. All these provisions could create a situation where O_2_ is the rate‐limiting substrate for the electron transport chain, which could explain our observation that post‐arrest VO_2_ is O_2_ concentration dependent.

### Study Limitations

This study is subject to several limitations. The first is that we studied post‐arrest metabolism in rats. The novel findings reported in this article may not be found in humans.^61^ We are currently conducting human studies to seek this newly found phenotype in survivors of CA. Second, the control group, the sham‐FIO_2_ 0.3 group, has 2 differences relative to the CA groups: it is missing the injury and it includes isoflurane, which is absent in the other groups after successful resuscitation. It is plausible that isoflurane alters the systemic metabolism in rodents. However, our VO_2_, VCO_2_, and RQ values in the sham‐FIO_2_ 0.3 group are within a range reported for other rat models in the absence of isoflurane.^36^, ^62^ This suggests that the CA injury is solely responsible for the observed changes in RQ values. Finally, our volume measurements may underestimate the true tidal volume. It is possible that there was a small amount of leakage around the uncuffed endotracheal tube used in these studies. In addition, we used a side‐stream capnograph and its sampling rate was set at 10 mL/min. However, our baseline VO_2_ and VCO_2_ values fall into the previously reported range, and we see no evidence that the CA injury had any additional effect on tidal volumes or airway pressures.

## Conclusions

We provide real‐time, in vivo evidence of a novel postresuscitation metabolic phenotype in resuscitated rats measured by indirect calorimetry. The phenotype is characterized by a 50% to 100% increase in VO_2_ without a concomitant increase in VCO_2_. As such, the metabolic phenotype is not a change in metabolic rate but a fundamental shift in cellular O_2_ utilization. There are several possible biochemical explanations for this phenomenon, but they remain untested. Our findings may be relevant to basic scientists who study O_2_ metabolism and cellular biochemistry, as well as to clinicians who seek better monitoring technologies and therapies for the treatment of CA.

## Sources of Funding

The research reported in this publication was supported by the National Heart, Lung, and Blood Institute of the National Institutes of Health; under the award number RO1HL67630 (Becker). The content is solely the responsibility of the authors and does not necessarily represent the official views of the National Institutes of Health.

## Disclosures

Saeki is an employee of Nihon Kohden Innovation Center, INC. There are no products in development or marketed products to declare. This does not alter the authors’ adherence to all of the Journal's policies on sharing data and materials. Shinozaki, Lampe, and Becker own intellectual property in metabolic measurements in critically ill patients. Shinozaki has grant/research support from Nihon Kohden Corp. Lampe has grant/research support from Zoll Medical Corp., Philips Healthcare, Nihon Kohden Corp., and the National Institutes of Health, and owns intellectual property in resuscitation devices. Becker has grant/research support from Philips Healthcare, the National Institutes of Health, Nihon Kohden Corp., BeneChill Inc., Zoll Medical Corp, and Medtronic Foundation, and patents in the areas of hypothermia induction and perfusion therapies. The remaining authors have no disclosures to report.
